# Supplementary material for: Trans Isomeric Fatty Acids in Children and Young Adults with Type 1 Diabetes Mellitus
Source: Nutrients. 2025 Jun 1;17(11):1907. doi: 10.3390/nu17111907 (PMC12157030; doi:10.3390/nu17111907)
Supplement: Supplementary file 1 [file nutrients-17-01907-s001.zip › nutrients-3577016-supplementary.pdf]

Supplementary files

Supplementary Table S1. Characteristics of the subjects.

	Young adults				Children				Diabetic children with DKA at admission	
	T1DM (n = 34)		control (n = 36)		T1DM (n = 40)		control (n = 40)		(n = 9)	
	mean	SD	mean	SD	mean	SD	mean	SD	mean	SD
Gender (male:female)	19:15		19:17		12:28		18:22		4:5	
Age (years)	21.77	3.09	22.63	2.47	12.03	3.90	12.45	3.49	16.71	4.27
Weight (kg)	68.10	11.83	66.00	19.00	44.55	16.74	44.76	14.71	56.50	27.00
BMI (kg/m^2)	22.70	5.32	21.93	2.56	19.35	3.61	18.43	3.53	19.50	8.00
Cholesterol (mmol/l)	4.72	0.96	4.55	0.72	5.99	6.55	4.15	0.94		
Triglyceride (mmol/l)	1.20	0.89	0.97	0.40	1.16	0.81	0.92	0.37		
HbA1C (%)	8.86	2.77	4.75	0.34	9.24	2.29	5.29	0.47	13.40	2.80
Duration of T1DM (years)	11.37	5.78			6.30	3.48			6.44	7.08
Daily insulin dose (U/kg/day)	0.76	0.30			1.05	0.86			0.87	0.66

Abbreviations denote: BMI: body mass index, DKA: diabetic ketoacidosis, HbA1C: haemoglobin A1C, SD: standard deviation, T1DM: type 1 diabetes mellitus

Supplementary Table S2: Correlation coefficients between *trans* isomeric fatty acids and n-6 polyunsaturated fatty acids in diabetic young adults (n = 34) and controls (n = 36) in plasma and erythrocyte membrane lipid fractions.

	C16:1n-7*t*		C18:1n-7/9*t*		C18:2n-6*tt*		Sumtrans	
	Diabetes	Control	Diabetes	Control	Diabetes	Control	Diabetes	Control
**C18:2n-6**								
**PL**	-0.207	-0.033	0.149	-0.242	0.229	0.089	0.098	-0.106
**TG**	-0.097	0.135	0.100	-0.303	-0.007	-0.132	0.039	-0.268
**STE**	**-0.449****	-0.239	0.156	-0.061	-0.041	**0.420***	**-0.389***	-0.090
**NEFA**	-0.285	**0.554***	**0.528****	**-0.474***	**0.426***	0.209	**0.357***	0.152
**PC**	-0.257	0.125	-0.218	0.068	-0.001	-0.077	-0.228	0.024
**PE**	0.053	0.041	0.307	0.175	0.262	-0.011	0.338	0.160
**C20:3n-6**								
**PL**	0.163	-0.098	-0.066	-0.170	**0.358***	-0.214	0.211	-0.158
**TG**	**0.588*****	0.265	0.285	0.199	0.276	-0.171	**0.449****	**0.343***
**STE**	**0.640*****	**0.395***	0.107	0.256	0.062	0.251	**0.579*****	**0.420***
**NEFA**	**0.414***	0.018	-0.002	0.298	**0.426***	0.159	0.193	0.026
**PC**	0.253	0.008	**0.464****	-0.097	0.218	0.099	**0.502****	-0.059
**PE**	**0.391***	0.222	0.144	0.203	0.140	-0.072	0.242	0.146
**C20:4n-6**								
**PL**	-0.113	-0.277	**-0.400***	-0.275	-0.174	-0.094	**-0.380***	-0.244
**TG**	0.070	0.181	-0.315	-0.062	-0.326	-0.034	-0.267	0.091
**STE**	**0.371***	-0.140	-0.286	-0.288	-0.188	-0.199	0.204	-0.267
**NEFA**	**0.477****	0.059	-0.021	0.117	0.244	0.401	0.221	-0.226
**PC**	-0.011	**-0.395***	0.296	**-0.329***	0.147	0.056	0.279	**-0.331***
**PE**	-0.291	0.159	**-0.441***	0.266	-0.118	0.019	**-0.451****	0.247
**C22:4n-6**								
**PL**	0.121	-0.089	-0.142	-0.025	-0.115	-0.063	-0.127	0.072
**TG**	-0.082	0.069	-0.277	**0.462****	-0.310	0.109	-0.295	**0.517****
**STE**	0.121	0.288	-0.142	0.361	-0.115	**0.384***	-0.127	**0.422***
**NEFA**	-0.099	**0.707****	-0.343	-0.236	**-0.501****	-0.067	**-0.389***	0.439
**PC**	-0.099	-0.312	**0.342***	-0.175	0.184	0.205	0.283	-0.170
**PE**	-0.219	0.290	-0.038	0.170	0.023	0.224	-0.060	0.216
**C22:5n-6**								
**PL**	-0.126	-0.166	-0.029	-0.001	-0.214	-0.304	-0.018	-0.102
**TG**	0.178	-0.171	0.295	0.264	-0.031	-0.265	0.266	0.179
**STE**	-0.126	-0.182	-0.029	0.126	-0.214	-0.034	-0.018	-0.056
**NEFA**	-0.115	0.473	-0.038	-0.213	0.033	-0.350	-0.055	0.248
**PC**	-0.134	**-0.336***	0.274	**-0.358***	0.156	0.101	0.231	**-0.364***
**PE**	-0.049	0.159	-0.095	0.160	0.113	0.056	-0.102	0.111
**n-6 PUFA**								
**PL**	**-0.385***	-0.159	-0.032	**-0.349***	0.195	-0.059	-0.152	-0.237
**TG**	-0.045	0.160	0.040	-0.238	-0.089	-0.127	0.004	-0.192
**STE**	**-0.385***	-0.183	-0.032	-0.110	0.195	**0.363***	-0.152	-0.103
**NEFA**	-0.183	**0.587***	**0.431***	**-0.509***	**0.417***	0.187	0.304	0.117
**PC**	-0.315	**-0.350***	0.156	-0.150	0.187	-0.027	0.103	-0.222

**PE**	-0.323	0.238	-0.005	0.312	0.105	0.120	-0.030	0.301
**n-6 LCPUFA**								
**PL**	-0.121	-0.257	-0.315	-0.320	-0.107	-0.261	-0.272	-0.311
**TG**	0.276	0.214	-0.167	0.107	-0.235	0.013	-0.081	0.273
**STE**	-0.121	0.058	-0.315	-0.150	-0.107	-0.110	-0.272	-0.065
**NEFA**	0.283	0.152	-0.228	0.077	0.015	0.253	-0.065	-0.100
**PC**	0.002	**-0.375***	**0.357***	-0.312	0.144	0.110	0.332	-0.302
**PE**	-0.240	0.226	-0.292	0.298	-0.055	0.121	-0.298	0.278

Abbreviations denote: n-6 LCPUFA: sum of all n-6 long chain polyunsaturated fatty acids; n-6 PUFA: sum of all n-6 polyunsaturated fatty acids; NEFA: non esterified fatty acids; PC: phosphatidylcholine; PE: phosphatidylethanolamine; PL: phospholipid; STE: sterol ester; TG: triacylglycerol

Bold numbers indicate significant correlation * at $p < 0.05$; ** at $p < 0.01$; *** at $p < 0.001$

Light orange background indicates significant negative correlations, light blue background significant positive correlations.

Supplementary Table S3: Correlation coefficients between *trans* isomeric fatty acids and n-3 polyunsaturated fatty acids in diabetic young adults (n = 34) and controls (n = 36) in plasma and erythrocyte membrane lipid fractions.

	C16:1n-7*t*		C18:1n-7/9*t*		C18:2n-6*tt*		Sumtrans	
	Diabetes	Control	Diabetes	Control	Diabetes	Control	Diabetes	Control
**C18:3n-3**								
**PL**	0.165	-0.246	**0.385***	-0.074	-0.043	-0.180	0.268	-0.228
**TG**	-0.080	-0.141	0.148	0.302	**0.341***	-0.038	0.104	0.170
**STE**	**0.675*****	0.268	-0.130	0.119	0.077	0.255	**0.557*****	**0.340***
**NEFA**	-0.195	-0.323	0.350	0.337	0.268	0.099	0.260	-0.077
**PC**	-0.195	0.243	0.350	0.063	0.268	0.012	0.260	0.073
**PE**	0.231	0.218	0.118	**0.608*****	-0.285	0.035	0.161	**0.563*****
**C20:5n-3**								
**PL**	0.087	-0.018	-0.165	-0.092	-0.001	0.177	-0.159	0.003
**TG**	0.329	0.069	-0.011	0.088	0.072	0.135	0.108	0.183
**STE**	**0.406***	0.137	-0.128	-0.083	**0.416***	0.108	0.331	0.094
**NEFA**	0.566	0.170	0.175	0.000	0.231	0.233	0.517	0.126
**PC**	**0.619*****	0.004	0.320	**0.377***	-0.069	0.108	**0.390***	0.303
**PE**	0.175	-0.127	-0.318	0.193	-0.211	-0.176	-0.197	0.125
**C22:5n-3**								
**PL**	-0.005	-0.064	**-0.480****	-0.091	-0.180	0.176	**-0.396***	0.039
**TG**	**-0.595*****	0.090	**-0.466****	0.204	**-0.516****	0.042	**-0.553*****	0.247
**STE**	-0.200	**0.483****	0.336	**0.517****	0.246	-0.017	-0.053	**0.500****
**NEFA**	0.213	0.571	-0.045	-0.548	0.078	**-0.829***	0.029	**0.810***
**PC**	0.096	-0.233	0.328	-0.041	0.329	0.114	**0.343***	-0.007
**PE**	0.136	-0.053	-0.063	0.229	-0.100	-0.095	0.012	0.201
**C22:6n-3**								
**PL**	-0.169	-0.052	0.023	-0.180	-0.267	-0.087	-0.046	-0.144
**TG**	0.026	0.127	-0.101	-0.121	-0.222	0.032	-0.088	-0.006
**STE**	0.158	-0.295	0.012	-0.190	0.211	-0.128	0.192	-0.268
**NEFA**	**0.515****	0.063	0.220	0.042	0.346	0.451	**0.440***	-0.001
**PC**	-0.079	**-0.512****	0.123	-0.137	0.101	0.098	0.096	-0.181
**PE**	-0.036	**-0.450****	**-0.380***	-0.089	-0.102	-0.113	-0.310	-0.232
**n-3 PUFA**								
**PL**	-0.079	-0.092	-0.044	-0.246	-0.273	-0.045	-0.117	-0.179
**TG**	-0.241	0.036	-0.126	0.261	-0.149	-0.014	-0.168	0.297
**STE**	**0.525****	0.219	-0.064	0.105	**0.456****	0.188	**0.489****	0.284
**NEFA**	**0.537****	0.280	0.346	-0.214	**0.465****	0.181	**0.598*****	0.077
**PC**	0.070	**-0.452****	0.240	-0.087	0.108	0.068	0.234	-0.126
**PE**	0.082	-0.317	**-0.365***	0.106	-0.143	-0.159	-0.253	-0.026
**n-3 LCPUFA**								
**PL**	-0.119	-0.099	-0.088	-0.231	-0.208	-0.054	-0.130	-0.167
**TG**	-0.203	0.073	-0.336	0.138	**-0.386***	-0.045	-0.334	0.225
**STE**	**0.370***	-0.005	-0.041	-0.024	**0.450****	-0.022	**0.368***	0.025
**NEFA**	**0.577*****	0.302	0.208	-0.127	**0.365***	0.209	**0.448***	0.174
**PC**	0.070	**-0.459****	0.248	-0.087	0.101	0.064	0.242	-0.130
**PE**	0.076	**-0.340***	**-0.367***	0.055	-0.113	-0.176	-0.256	-0.075

Abbreviations denote: n-3 LCPUFA: sum of all n-3 long chain polyunsaturated fatty acids; n-3 PUFA: sum of all n-3 polyunsaturated fatty acids; NEFA: non esterified fatty acids; PC: phosphatidylcholine; PE: phosphatidylethanolamine; PL: phospholipid; STE: sterol ester; TG: triacylglycerol

Bold numbers indicate significant correlation * at $p < 0.05$; ** at $p < 0.01$; *** at $p < 0.001$

Light orange background indicates significant negative correlations, light blue background significant positive correlations.

Supplementary Table S4: Correlation coefficients between *trans* isomeric fatty acids and n-6 polyunsaturated fatty acids in diabetic children (n = 40) and controls (n = 40) in plasma and erythrocyte membrane lipid fractions.

	**C16:1n-7*t***		**C18:1n-7/9*t***		**C18:2n-6*tt***		**Sumtrans**	
	**Diabetes**	**Control**	**Diabetes**	**Control**	**Diabetes**	**Control**	**Diabetes**	**Control**
**C18:2n-6**								
**PL**	-0.259	0.028	0.093	-0.101	-0.104	**0.439****	0.032	-0.049
**TG**	0.125	0.059	-0.262	-0.202	**-0.359***	**-0.330***	-0.197	-0.175
**STE**	**-0.496****	**-0.443****	-0.154	**-0.442****	**-0.509****	-0.017	**-0.374***	**-0.562*****
**NEFA**	-0.049	**-0.376***	**0.502*****	**0.571*****	0.078	-0.183	**0.377***	0.293
**PC**	-0.014	0.198	-0.012	0.023	-0.237	-0.190	-0.054	0.073
**PE**	0.319	0.262	0.197	-0.002	-0.092	0.255	0.267	0.116
**C20:3n-6**								
**PL**	**0.430****	**0.341***	**0.318***	0.038	-0.232	0.105	**0.350***	0.060
**TG**	**0.581*****	**0.529*****	-0.047	0.005	-0.002	0.112	0.313	0.198
**STE**	0.310	**0.421****	**-0.346***	-0.212	0.019	**-0.379***	-0.030	0.070
**NEFA**	0.301	0.180	0.068	0.306	0.126	0.032	0.118	**0.331***
**PC**	0.195	0.285	0.136	0.130	-0.074	0.276	0.078	0.194
**PE**	0.055	0.217	0.276	0.265	-0.012	0.292	0.274	**0.353***
**C20:4n-6**								
**PL**	0.125	-0.135	-0.122	-0.253	-0.126	**-0.465****	-0.110	-0.290
**TG**	0.228	-0.223	-0.250	**-0.312***	-0.055	**-0.364***	-0.098	**-0.325***
**STE**	-0.072	-0.156	-0.073	**-0.623*****	-0.092	**-0.356***	-0.133	**-0.510*****
**NEFA**	0.279	**0.400***	-0.174	0.044	**0.330***	-0.212	-0.037	0.191
**PC**	**-0.348***	-0.253	-0.021	-0.161	0.276	0.097	-0.063	-0.154
**PE**	0.266	**0.400***	-0.049	-0.309	-0.234	-0.064	0.003	-0.050
**C22:4n-6**								
**PL**	-0.040	0.225	0.239	0.155	0.168	0.050	0.253	0.147
**TG**	**0.336***	-0.054	0.084	-0.105	**0.464****	-0.086	**0.329***	-0.074
**STE**	-0.136	0.187	-0.231	**-0.411****	-0.213	-0.325	-0.250	-0.127
**NEFA**	-0.155	0.114	-0.261	-0.162	**0.538*****	0.230	-0.109	-0.022
**PC**	-0.241	-0.219	-0.028	0.030	0.238	0.231	-0.061	0.051
**PE**	-0.015	0.146	-0.001	0.132	0.008	0.259	0.007	0.159
**C22:5n-6**								
**PL**	0.110	0.220	**0.328***	0.193	0.016	-0.138	**0.316***	0.150
**TG**	0.209	0.071	-0.135	-0.130	-0.051	0.044	0.005	-0.037
**STE**	-0.033	0.200	-0.099	0.079	0.134	0.151	0.070	0.169
**NEFA**	0.312	0.240	-0.327	-0.088	-0.018	0.213	-0.269	0.120
**PC**	-0.257	-0.188	-0.057	-0.030	0.305	0.185	-0.055	-0.007
**PE**	-0.237	0.267	0.108	0.044	0.089	0.203	0.024	0.186
**n-6 PUFA**								
**PL**	-0.060	0.017	0.194	-0.172	-0.238	0.154	0.143	-0.143
**TG**	0.209	0.029	-0.282	-0.237	**-0.346***	**-0.334***	-0.167	-0.206
**STE**	**-0.527*****	**-0.501*****	-0.220	**-0.673*****	**-0.574*****	-0.234	**-0.461****	**-0.773*****
**NEFA**	-0.004	-0.283	**0.442****	**0.538*****	0.135	-0.169	**0.349***	**0.336***
**PC**	-0.295	-0.175	0.038	-0.133	0.034	0.051	-0.036	-0.097

**PE**	0.313	**0.465****	0.101	-0.262	-0.208	0.163	0.163	-0.002
**n-6 LCPUFA**								
**PL**	0.300	0.038	0.053	-0.159	-0.212	**-0.406***	0.070	-0.192
**TG**	**0.444****	-0.029	-0.203	-0.301	-0.025	**-0.326***	0.057	-0.250
**STE**	-0.030	-0.110	-0.070	**-0.633*****	-0.072	**-0.411***	-0.098	**-0.494****
**NEFA**	0.247	**0.379***	-0.113	-0.034	**0.349***	-0.081	0.014	0.214
**PC**	-0.299	-0.238	-0.024	-0.122	0.230	0.177	-0.070	-0.107
**PE**	0.147	**0.417****	0.002	-0.194	-0.181	0.133	0.026	0.039

Abbreviations denote: n-6 LCPUFA: sum of all n-6 long chain polyunsaturated fatty acids; n-6 PUFA: sum of all n-6 polyunsaturated fatty acids; NEFA: non esterified fatty acids; PC: phosphatidylcholine; PE: phosphatidylethanolamine; PL: phospholipid; STE: sterol ester; TG: triacylglycerol

Bold numbers indicate significant correlation * at $p < 0.05$; ** at $p < 0.01$; *** at $p < 0.001$

Light orange background indicates significant negative correlations, light blue background significant positive correlations.

Supplementary Table S5: Correlation coefficients between *trans* isomeric fatty acids and n-3 polyunsaturated fatty acids in diabetic children (n = 40) and controls (n = 40) in plasma and erythrocyte membrane lipid fractions.

	C16:1n-7*t*		C18:1n-7/9*t*		C18:2n-6*tt*		Sumtrans	
	**Diabetes**	**Control**	**Diabetes**	**Control**	**Diabetes**	**Control**	**Diabetes**	**Control**
**C18:3n-3**								
**PL**	0.074	0.017	0.112	0.278	0.249	-0.033	0.174	0.238
**TG**	0.074	0.257	0.112	0.210	0.249	0.289	0.174	0.225
**STE**	0.129	0.295	0.074	**-0.323***	0.267	-0.095	0.109	0.088
**NEFA**	0.030	-0.027	**0.347***	**0.335***	0.188	0.206	**0.349***	**0.447***
**PC**	0.072	0.122	0.256	0.317	0.061	-0.096	0.217	0.255
**PE**	0.285	0.104	0.107	-0.032	-0.177	0.163	0.184	0.047
**C20:5n-3**								
**PL**	0.209	-0.068	-0.030	0.032	0.052	0.028	0.036	0.042
**TG**	0.209	0.224	-0.030	**0.569*****	0.052	**0.409***	0.036	**0.568*****
**STE**	0.102	0.251	0.009	**-0.388***	0.064	-0.098	0.008	-0.072
**NEFA**	-0.014	0.251	0.087	0.083	0.260	0.144	0.108	0.320
**PC**	0.163	-0.048	-0.007	0.124	0.000	-0.032	0.060	0.082
**PE**	-0.062	-0.072	0.027	0.124	0.286	0.047	0.078	0.131
**C22:5n-3**								
**PL**	0.079	-0.028	-0.130	0.159	-0.048	0.194	-0.121	0.185
**TG**	0.172	0.088	**0.456****	**0.355***	**0.359***	**0.624*****	**0.468****	**0.451****
**STE**	-0.215	0.102	-0.308	-0.290	-0.293	-0.319	**-0.405***	-0.157
**NEFA**	**0.320***	0.250	-0.201	-0.118	-0.164	-0.017	-0.063	-0.027
**PC**	-0.252	-0.219	0.080	0.032	0.101	0.310	0.018	0.050
**PE**	-0.297	**-0.340***	-0.053	0.309	**0.451***	0.311	-0.037	0.211
**C22:6n-3**								
**PL**	-0.046	**-0.332***	-0.284	-0.029	0.077	0.094	-0.266	-0.023
**TG**	-0.212	-0.124	0.087	0.121	0.239	0.194	-0.064	0.116
**STE**	0.002	-0.142	0.148	**-0.536*****	0.178	-0.242	0.158	**-0.391***
**NEFA**	0.168	0.130	-0.026	-0.018	**0.470****	**0.410***	0.136	0.133
**PC**	-0.284	-0.319	-0.007	-0.090	0.258	0.275	-0.022	-0.082
**PE**	0.181	-0.088	0.140	-0.189	0.080	0.102	0.179	-0.077
**n-3 PUFA**								
**PL**	-0.111	-0.274	-0.269	0.030	0.196	0.095	-0.231	0.041
**TG**	0.038	-0.032	**0.462****	0.290	0.251	**0.395***	**0.357***	0.230
**STE**	0.050	0.170	-0.019	**-0.561*****	0.185	-0.239	0.026	-0.201
**NEFA**	0.286	0.215	0.147	0.186	**0.351***	**0.420****	0.304	**0.387***
**PC**	-0.256	-0.301	0.021	-0.049	0.241	0.277	0.000	-0.043
**PE**	-0.010	-0.241	0.065	-0.004	0.285	0.094	0.108	0.042
**n-3 LCPUFA**								
**PL**	-0.100	-0.286	-0.254	-0.007	0.158	0.073	-0.224	0.003
**TG**	-0.035	-0.058	**0.337***	0.248	**0.431****	**0.384***	0.240	0.241
**STE**	0.070	0.072	0.075	**-0.505*****	0.137	-0.250	0.083	-0.257
**NEFA**	0.265	0.213	-0.144	-0.014	0.288	0.285	0.033	0.128
**PC**	-0.253	-0.299	0.018	-0.048	0.254	0.283	0.003	-0.040
**PE**	-0.020	-0.225	0.052	0.002	0.315	0.074	0.088	0.050

Abbreviations denote: n-3 LCPUFA: sum of all n-3 long chain polyunsaturated fatty acids; n-3 PUFA: sum of all n-3 polyunsaturated fatty acids; NEFA: non esterified fatty acids; PC: phosphatidylcholine; PE: phosphatidylethanolamine; PL: phospholipid; STE: sterol ester; TG: triacylglycerol

Bold numbers indicate significant correlation * at $p < 0.05$; ** at $p < 0.01$; *** at $p < 0.001$

Light orange background indicates significant negative correlations, light blue background significant positive correlations.

Supplementary Table S6: Correlation coefficients between *trans* isomeric fatty acids and n-6 polyunsaturated fatty acids in diabetic children during and after diabetic ketoacidosis (DKA) in plasma lipid fractions.

	C16:1n-7*t*		C18:1n-7/9*t*		C18:2n-6*tt*		Sumtrans	
	During DKA	After DKA	During DKA	After DKA	During DKA	After DKA	During DKA	After DKA
**C18:2n-6**								
**PL**	0.367	-0.117	-0.450	**-0.700***	-0.167	0.083	-0.450	-0.367
**TG**	-0.567	-0.117	**-0.683***	-0.550	-0.400	0.083	**-0.767***	-0.333
**STE**	-0.033	-0.400	-0.317	-0.267	-0.200	-0.067	-0.100	-0.250
**NEFA**	0.017	**-0.733***	-0.217	**-0.717***	-0.033	0.433	-0.017	**-0.750***
**C20:3n-6**								
**PL**	-0.150	0.600	-0.183	-0.050	-0.433	-0.217	-0.183	0.033
**TG**	-0.017	0.200	0.650	0.367	0.367	0.500	0.583	0.617
**STE**	0.383	0.233	-0.133	0.100	-0.383	-0.400	-0.017	0.117
**NEFA**	0.433	0.267	0.317	**0.750***	0.067	0.050	0.300	0.600
**C20:4n-6**								
**PL**	-0.567	-0.117	-0.583	**-0.733***	-0.467	-0.483	-0.583	**-0.867****
**TG**	-0.383	0.500	-0.417	-0.567	**-0.733***	-0.300	-0.433	-0.500
**STE**	-0.433	0.017	-0.617	-0.417	**-0.767***	**-0.750***	**-0.733***	-0.300
**NEFA**	0.433	0.167	0.267	**0.667***	0.150	0.167	0.400	0.467
**C22:4n-6**								
**PL**	0.133	0.217	-0.433	0.367	-0.400	-0.167	-0.433	0.267
**TG**	-0.317	0.567	**-0.717***	-0.183	-0.633	-0.283	**-0.667***	-0.200
**STE**	0.133	0.300	-0.433	0.283	-0.250	-0.233	-0.333	0.317
**NEFA**	0.550	0.150	0.583	**0.750***	-0.067	0.033	0.567	0.617
**C22:5n-6**								
**PL**	-0.283	-0.183	-0.600	-0.417	**-0.767***	-0.483	-0.600	-0.600
**TG**	0.317	0.333	0.350	0.267	0.150	-0.267	0.417	0.067
**STE**	-0.133	0.367	0.083	0.567	0.183	**0.683***	0.200	0.500
**NEFA**	0.283	-0.400	-0.167	-0.033	0.400	0.250	0.117	-0.167
**n-6 PUFA**								
**PL**	0.183	-0.050	**-0.733***	**-0.900****	-0.217	-0.217	**-0.733***	**-0.700***
**TG**	-0.567	-0.083	**-0.683***	-0.467	-0.400	0.150	**-0.767***	-0.250
**STE**	-0.117	-0.100	-0.333	-0.217	-0.467	-0.300	-0.200	-0.100
**NEFA**	**0.667***	-0.650	0.200	-0.267	0.433	0.367	**0.700***	-0.367
**n-6 LCPUFA**								
**PL**	-0.567	-0.133	-0.583	-0.533	-0.467	-0.550	-0.583	**-0.750***
**TG**	-0.567	-0.083	**-0.683***	-0.467	-0.400	0.150	**-0.767***	-0.250
**STE**	-0.300	0.017	-0.600	-0.417	**-0.783***	**-0.750***	**-0.717***	-0.300
**NEFA**	0.617	0.233	0.400	**0.750***	0.017	0.067	0.600	0.617

Abbreviations denote: n-6 LCPUFA: sum of all n-6 long chain polyunsaturated fatty acids; n-6 PUFA: sum of all n-6 polyunsaturated fatty acids; NEFA: non esterified fatty acids; PL: phospholipid; STE: sterol ester; TG: triacylglycerol

Bold numbers indicate significant correlation * at $p < 0.05$; ** at $p < 0.01$

Light orange background indicates significant negative correlations, light blue background significant positive correlations.

Supplementary Table S7: Correlation coefficients between *trans* isomeric fatty acids and n-3 polyunsaturated fatty acids in diabetic children (n = 9) during and after diabetic ketoacidosis (DKA) in plasma lipid fractions.

	C16:1n-7*t*		C18:1n-7/9*t*		C18:2n-6*tt*		Sumtrans	
	During DKA	After DKA	During DKA	After DKA	During DKA	After DKA	During DKA	After DKA
**C18:3n-3**								
**PL**	-0.617	-0.533	-0.517	0.183	-0.333	0.583	-0.517	0.300
**TG**	0.017	-0.367	**-0.733***	-0.550	**-0.750***	-0.617	**-0.683***	**-0.683***
**STE**	0.483	0.067	0.267	0.050	-0.217	0.267	0.333	0.117
**NEFA**	-0.300	-0.217	-0.500	**-0.783***	0.250	-0.050	-0.367	**-0.667***
**C20:5n-3**								
**PL**	**-0.733***	0.533	-0.450	0.083	-0.367	-0.150	-0.450	0.100
**TG**	0.250	0.300	-0.267	-0.233	-0.467	-0.517	-0.300	-0.383
**STE**	0.150	-0.033	-0.150	0.200	-0.500	0.100	-0.217	0.150
**NEFA**	0.350	0.383	-0.033	0.283	**0.717***	-0.550	0.350	0.317
**C22:6n-3**								
**PL**	-0.383	0.233	-0.517	-0.167	-0.067	0.250	-0.517	0.033
**TG**	0.067	0.350	-0.267	-0.033	-0.417	0.083	-0.283	0.017
**STE**	-0.483	0.417	-0.467	0.167	-0.150	0.267	-0.367	0.333
**NEFA**	0.033	0.617	0.067	0.400	0.300	-0.417	0.133	0.567
**n-3 PUFA**								
**PL**	-0.283	0.267	-0.333	-0.300	-0.133	0.133	-0.333	-0.117
**TG**	0.167	0.267	-0.600	-0.417	**-0.683***	-0.500	-0.583	-0.483
**STE**	-0.067	0.167	-0.133	0.250	-0.333	0.250	-0.050	0.283
**NEFA**	-0.083	0.117	0.133	-0.317	**0.783***	**-0.850****	0.333	-0.167
**n-3 LCPUFA**								
**PL**	-0.267	0.300	-0.233	-0.200	-0.033	0.150	-0.233	-0.033
**TG**	0.350	0.067	-0.333	-0.617	-0.450	**-0.800****	-0.300	**-0.800****
**STE**	-0.167	0.150	-0.033	0.367	-0.300	0.200	0.017	0.367
**NEFA**	0.267	0.417	0.100	0.317	0.267	-0.600	0.300	0.383

Abbreviations denote: n-3 LCPUFA: sum of all n-3 long chain polyunsaturated fatty acids; n-3 PUFA: sum of all n-3 polyunsaturated fatty acids; NEFA: non esterified fatty acids; PL: phospholipid; STE: sterol ester; TG: triacylglycerol

Bold numbers indicate significant correlation * at $p < 0.05$; ** at $p < 0.01$

Light orange background indicates significant negative correlations, light blue background significant positive correlations.
